# A Super-Enhancer Driven by FOSL1 Controls miR-21-5p Expression in Head and Neck Squamous Cell Carcinoma

**DOI:** 10.3389/fonc.2021.656628

**Published:** 2021-04-16

**Authors:** Yuehan Wan, Rosalie G. Hoyle, Nan Xie, Wenjin Wang, Hongshi Cai, Ming Zhang, Zhikun Ma, Gan Xiong, Xiuyun Xu, Zhengxian Huang, Xiqiang Liu, Jiong Li, Cheng Wang

**Affiliations:** ^1^ Department of Oral and Maxillofacial Surgery, Hospital of Stomatology, Guanghua School of Stomatology, Sun Yat-Sen University, Guangzhou, China; ^2^ Guangdong Provincial Key Laboratory of Stomatology, Sun Yat-Sen University, Guangzhou, China; ^3^ Department of Medicinal Chemistry, Institute for Structural Biology, Drug Discovery and Development, School of Pharmacy and the Massey Cancer Center, Virginia Commonwealth University, Richmond, VA, United States; ^4^ Department of Oral Pathology, Guanghua School of Stomatology, Hospital of Stomatology, Sun Yat-Sen University, Guangzhou, China; ^5^ Department of Oral and Maxillofacial Surgery, Nanfang Hospital, Southern Medical University, Guangzhou, China; ^6^ Department of Oral and Craniofacial Molecular Biology, Philips Institute for Oral Health Research, School of Dentistry, Virginia Commonwealth University, Richmond, VA, United States

**Keywords:** super enhancer, FOSL1, miR-21-5p, head and neck squamous cell carcinoma, metastasis

## Abstract

MiR-21-5p is one of the most common oncogenic miRNAs that is upregulated in many solid cancers by inhibiting its target genes at the posttranscriptional level. However, the upstream regulatory mechanisms of miR-21-5p are still not well documented in cancers. Here, we identify a super-enhancer associated with the *MIR21* gene (MIR21-SE) by analyzing the *MIR21* genomic regulatory landscape in head and neck squamous cell carcinoma (HNSCC). We show that the MIR21-SE regulates miR-21-5p expression in different HNSCC cell lines and disruption of MIR21-SE inhibits miR-21-5p expression. We also identified that a key transcription factor, FOSL1 directly controls miR-21-5p expression by interacting with the MIR21-SE in HNSCC. Moreover, functional studies indicate that restoration of miR-21-5p partially abrogates FOSL1 depletion-mediated inhibition of cell proliferation and invasion. Clinical studies confirmed that miR-21-5p expression is positively correlated with FOSL1 expression. These findings suggest that FOSL1-SE drives miR-21-5p expression to promote malignant progression of HNSCC

## Introduction

Head and neck squamous cell carcinoma (HNSCC) is one of the most common malignant tumors worldwide, with a 5-year survival rate of only about 50% (1, 2). HNSCC easily invades surrounding tissues and spreads to cervical lymph nodes (LN), ultimately leading to relapse and death (3). Although a significant progress on the pathogenesis of HNSCC has been made in the past decades, the exact molecular mechanisms are still not well understood. Increasing evidences implicates that dysregulation of miRNAs has a critical role in the development and progression of HNSCC (4–10). In our previous studies, we also confirmed that miRNAs are involved in the tumor growth, stemness and metastasis of HNSCC by targeting a cohort of key oncogenes or tumor suppressors, including miR-21, miR-204-5p, miR320a, miR-138 (11–13). Interestingly, the alterations of miRNA landscape showed that most of deregulated miRNA are downregulated in HNSCC as compared to normal tissue (14–17). Contrarily, only several upregulated miRNAs were identified, including miR-21-5p, miR-7 and miR-31 (18–22). Notably, miR-21-5p is one of the most common oncogenic miRNAs that is robustly upregulated and function as an oncogene in many solid cancers by inhibiting its target genes at the posttranscriptional level, including HNSCC (23, 24). However, the upstream regulatory mechanisms of miR-21 are still not well documented in cancers.

Herein, we aimed to investigate the upstream regulatory mechanisms of miR-21-5p in HNSCC. Strikingly, a MIR21-associated super enhancer (MIR21-SE) was identified in HNSCC and disruption of this super enhancer inhibits miR-21-5p expression. Interestingly, a key transcription factor, FOSL1, was confirmed to directly promote miR-21-5p expression by interacting with the MIR21-SE in HNSCC. Taken together, these findings showed that the miR-21-5p expression was controlled by a FOSL1-driving-SE in HNSCC.

## Materials and Methods

### Samples

95 OSCC tissues samples, 16 matched adjacent non-cancerous normal tissues (ANCT) were collected at the Department of Oral and Maxillofacial Surgery, Hospital of Stomatology, Sun Yat-sen University between January 2013 and July 2018. Tissue samples applied in this study were conducted in accordance with the Declaration of Helsinki’s guidelines. All patients did not receive any form of preoperative treatment. The study was approved by the Ethical Committee of the hospital. Tumor grade was determined according to the 8th American Joint Committee on Cancer Staging System. Immunohistochemical and *in situ* hybridization analysis were used to assess the gene expression.

### Immunohistochemistry and *In Situ* Hybridization

Formalin-fixed, paraffin-embedded tissue samples were cut into 4-μm sections for immunohistochemistry analysis. The tissue sections were incubated at 4°C with primary antibodies against FRA1/FOSL1(1:100, ab232745, Abcam) overnight. Diaminobenzidine (DAB, GK600510, Gene Tech, China) was used to visualize and hematoxylin (D006, Nanjing Jiancheng Biotech, China) was applied to counterstain. Two pathologists who were blinded to the clinical data evaluated the immunohistochemical stain independently. The intensity of FOSL1 staining was evaluated as 0: no staining; 1: weak; 2: moderate; and 3: strong. The proportion was evaluated as 0–100% positive cells. Staining index(SI: 0–300)=staining intensity × the proportion of positive cells. Samples with SI>100 were defined as “high expression” and those with SI ≤ 100 were defined as “low expression”.

miR-21-5p expression was examined by *in situ* hybridization according to the manufacturer’s instructions (microRNA ISH Optimization Kit for FFPE, Exiqon, Vedbaek, Denmark). miR-21-5p was hybridized with Double-DIG-labeled LNA™ microRNA probes (1:1250, Exiqon) overnight after demasking. The sections were blocked and incubated with goat anti-digoxigenin-AP (1:1000, Roche) and then counterstained with nitro blue tetrazolium/5-bromo-4-chloro-3-indolylphosphate (NBT/BCIP). The staining intensity of the cells was recorded as follows: The intensity staining was evaluated as 0: no staining; 1: weak, light blue; 2: moderate, blue; and 3: strong, dark blue. The proportion was evaluated as 0: negative; 1:<10%; 2: 11–50%; 3: 51–80% or 4: >80% positive cells. The staining index (SI)=staining intensity × the proportion of positive cells. SI (miR-21-5p)>4 was defined as “high expression” and SI ≤ 4 was defined as “low expression”.

### Chromatin Immunoprecipitation Assay (ChIP)

For ChIP assay, 10^6^ cells were treated with 1% formaldehyde at 37°C for 10 min to crosslink their DNA. Agarose gel electrophoresis was used to assess the DNA fragments between 500 bp and 800 bp the cells were lysed. The chromatin crosslink was incubated at 4°C overnight. Then purify the immunoprecipitated DNA after reversing the DNA–protein crosslink. Quantitative real-time PCR (qPCR) was used to quantify the final precipitated DNA. The PCR result was expressed with the percentage of input DNA. The primer sequences of ChIP-qPCR were listed as below. MIR21 super enhancer (MIR21-SE) 5’-AAACCACACTCTGTCGTATCTGTG-3’ and 5’-TACAGAACGGCAAGAAAACTGGG-3’ and negative control primer (MIR21-NEG) 5’- CCACCATGCCCAGCCTTGAAGTTA-3’ and 5’-TGGAGAGGGCTGACCTTAACCAA-3’.

### Luciferase Reporter Assay

MIR21-SE and MIR21-NEG fragments were inserted into pLG4.23 luciferase reporter through Kpn1 and Xho1 restriction sites using standard PCR-cloning method. The primer sequences for cloning MIR21-SE were 5’- GGGTACCGTTCTAGAAAAGAAGTGAAGGCCAGTCG-3’ and 5’- CCTCGAGTTAGACATGCTTGCAGGCGTTT-3’. The primer sequences for cloning MIR21-NEG were 5’- GGGTACCACCATGCCCAGCCTTGAAGTTAA-3’ and 5’- CCTCGAGACTGAGTGGGGAGAATTGCCTA -3’. For luciferase assay, SCC1 and 293T cells were plated in 24-well plates at 40-50% confluence. For luciferase assay in SCC1 cells, 12h after seeding, the cells were transfected with FOSL1 siRNA or control siRNA. Then, after 24h, the cells were transfected with 50 ng of luciferase reporters and 20 ng of CMV-galactosidase constructs. For luciferase assay in 293T cells, 12h after seeding, the cells were transfected with 50 ng of luciferase reporters, 20 ng of CMV-galactosidase, and expression constructs of FOSL1 and JUN. The reporter activities were determined 24h after DNA transfection. The luciferase and β-galactosidase activity of total cell lysates were determined by Bright-Glo Luciferase Assay System (Promega; cat#E2620) and GalactoStar Reporter Gene Assay System (Applied Biosystems, cat#T1012), respectively. The luciferase reporter activity was normalized against the β-galactosidase activity of each cell lysate sample.

### RNA Isolation and Quantitative Real-Time PCR (RT-qPCR)

RNAzol^®^ RT (RN190, Molecular Research Center, USA) was used to extracted RNA from the cells per the manufacturer’s protocol. 1 µg of total RNA was applied to reverse transcription with the ribo SCRIPT Reverse Transcription kit (C11027, RIBOBIO, China). All miR-21-5p (RT primter: ssD809230239; qPCR primer: ssD809230931 and ssD089261711) and U6 (RT primter: ssD0904071008; qPCR primer: ssD0904071006 and ssD0904071007) RT-qPCR primers were ordered from RIBOBIO. The 3-step RT-qPCR reactions were carried out with a SYBR Green Master Mix (11201ES08, Yeasen, China) in the LightCycler 96 System (Roche, Germany). The thermal cycling parameters were as follows: preincubation at 95°C for 5 min, followed by 40 cycles of amplification as: 95°C for 10 s, 60°C for 20 s, and 72°C for 20 s, with a final cycle of 95°C for 15 s, 60°C for 60 s, and 95°C for 15 s as the melting curve. The relative expression level of miR-21-5p was calculated by 2−ΔΔCt. Human U6 was served as an internal reference.

### Cell Lines, Cell Culture, and Treatments

SCC1 cells were obtained from University of Michigan and HN6 cells were obtained from Wayne State University. UM1 and UM2 cells were provided by Dr. Xiaofeng Zhou (University of Illinois at Chicago, IL, USA). SCC9, SCC15, SCC25 and CAL27 were obtained from the American Type Culture Collection (ATCC; Manassas, VA, USA). The SCC1, HN6 and CAL27 cell lines were cultivated in Dulbecco’s modified Eagle’s medium (DMEM, Gibco, USA) supplemented with 10% fetal bovine serum (FBS, Gibco, USA). The SCC9, SCC15, SCC25, UM1 and UM2 cell lines were grown in DMEM/F12 medium (DMEM/F12, Gibco, USA) supplemented with 10% FBS. Cells were kept at 37°C under a humidified atmosphere with 5% CO2. Lipofectamine (Lipofectamine RNAiMAX Transfection Reagent, Thermo Fisher) was used to transfect miR-21-5p mimics (50nM, RIBOBIO), FOSL1 siRNA (100nM, RIBOBIO) and their respective negative controls. For the BET bromodomain Inhibitor treatment groups, cells were treated with 1μmol/mL (+)-JQ1 (HY-13030, MCE, USA) or iBET-151 (HY-13235, MCE, USA).

### Cell Proliferation

HN6 and SCC1 cells were seeded in 96-well plates at a density of 1000 or 1500 cells per well. The cell confluence of untreated cells and treated cells were detected and analyzed using the Incucyte^®^ S3 Live-Cell Analysis System (Essen BioScience, USA) at the indicated time points.

### Wound-Healing, Migration, and Invasion Assays

Wound-healing assay was carried out with a sterile pipette tip to make scratches when cells reached 90% fusion degree in 6-well plates. FBS-free media was then used to cultivate cells for 48h. 2 × 10^5^ HN6 cells or 4 × 10^5^ SCC1 cells with 200μl FBS-free media were seeded into the upper chambers (pore size: 8-μm, Corning, China) with or without Matrigel (354234, Corning, China) to verify the invasion or migration assays. 600μl of 10% fetal bovine serum (FBS) was added into the lower chambers. Cells stranded in the upper chambers after 24h were removed, and the fixed cells on the lower surface of the membrane were stained with hematoxylin. Five random views were selected and photographed under a microscope (ZEISS, German). Image J was used to calculate the cell numbers and wound healing area.

### Statistical Analysis

GraphPad prism 8.0. (GraphPad Software Inc.) was used for statistical analysis with the mean ± standard deviation (SD). All statistical tests were two-tailed. P-values of <0.05 were considered statistically significant.

## Results

### miR-21-5p Is Upregulated and Correlated With Malignant Progression of HNSCC

**Figure 1:**
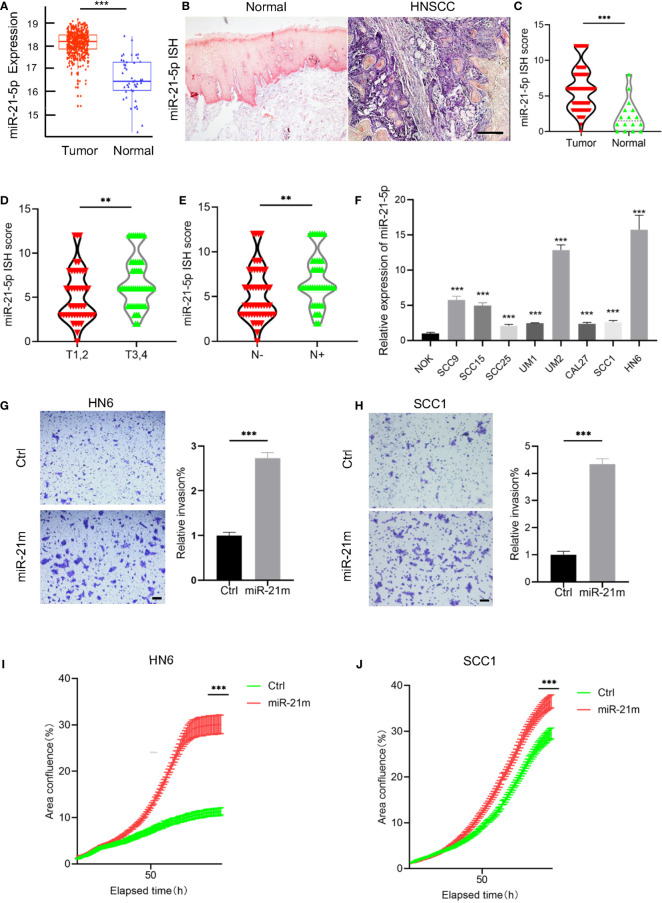
The expression of miR-21-5p was upregulated and correlated with malignant progression in HNSCC. **(A)** The expression of miR-21-5p was upregulated in HNSCC as compared to the normal tissue. Data was from TCGA HNSCC datasets downloaded using the UCSC Xena browser (https://xenabrowser.net). ***P < 0.001. **(B)** The representative images of miR-21-5p ISH staining. Scale bar, 300μm **(C)** The quantitative analysis of miR-21-5p ISH staining in HNSCC patient samples. ***P< 0.001 by Student’s t-test **(D)** The expression of miR-21-5p was increased in T3,4 stage HNSCC patient as comparted to those with T1,2 stage. **P < 0.01 by Student’s t-test **(E)** The expression of miR-21-5p was increased in HNSCC patient with lymph node metastasis as comparted to those without lymph node metastasis. **P < 0.01 by Student’s t-test **(F)** The relative of miR-21-5p was upregulated in 8 HNSCC cell lines as compared to NOK cells. ***P < 0.001 by Student t-test. **(G, H)** Overexpression of miR-21-5p promotes cell invasion of SCC1 and HN6 cells. ***P < 0.001 by Student’s t-test. Scale bar, 200μm **(I, J)** Overexpression of miR-21-5p promotes proliferation of SCC1 and HN6 cells. ***P < 0.001 by two-way ANOVA

To investigate the potential role of miR-21-5p in HNSCC, we firstly evaluated miR-21-5p expression based on The Cancer Genome Atlas (TCGA) HNSCC datasets. As shown in **Figure 1A**, the expression of miR-21-5p was significantly increased in HNSCC as compared to normal tissue. To further validate these findings, we performed *in situ* hybridization to detect the expression of miR-21-5p in 95 HNSCC tissues. As expected, we found that miR-21-5p expression was mainly located in cytoplasm and significantly increased in HNSCC when compared to normal epithelium (**Figures 1B, C** **)**. Moreover, the expression of miR-21-5p was also upregulated in HNSCC patient with T3,4 stage as compared to patients with T1,2 stage. Similar results were observed in HNSCC patient with lymph node metastasis as compared to patient without lymph node metastasis (**Figures 1D, E** **)**. An increase of miR-21-5p was also observed in an 8 HNSCC cell lines panel as compared to NOK cells (**Figure 1F**).

To further investigate the functional role miR-21-5p in HNSCC, HNSCC cells, HN6 and SCC1 were transfected with miR-21-5p mimics and we found that cell proliferation and invasion were increased in cells treated with miR-21-5p mimics **(** **Figures 1G–J** **)**. Taken together, these findings confirmed that miR-21-5p is upregulated in HNSCC and promotes malignant phenotypes in HNSCC.

### Targeting FOSL1 Suppresses miR-21-5p Expression by Interacting With the MIR21-SE

**Figure 2:**
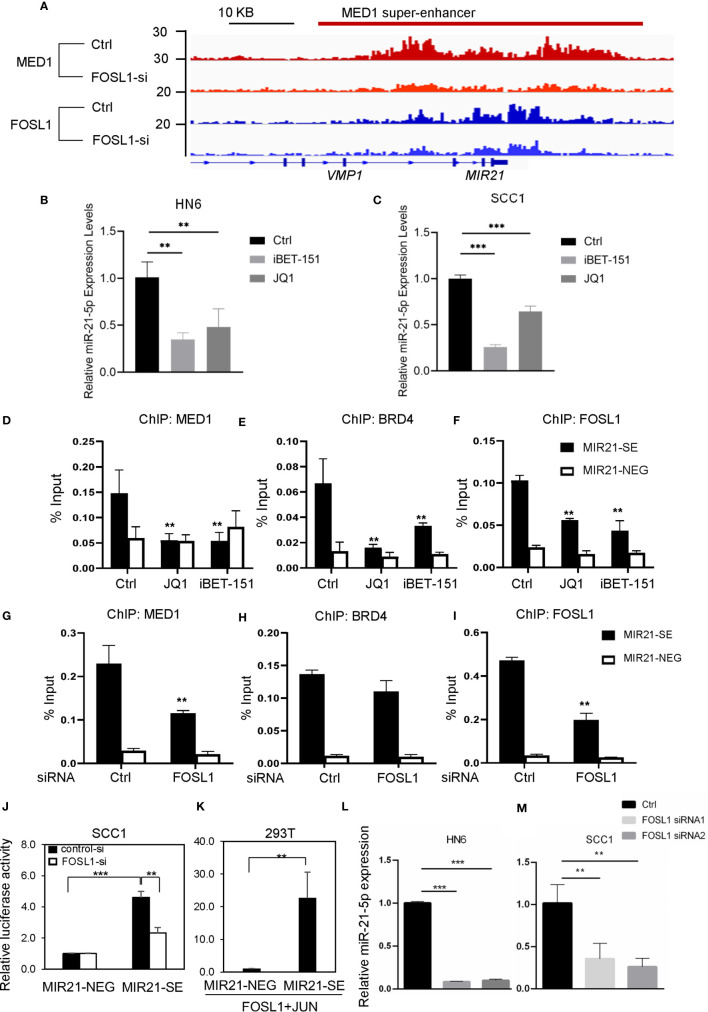
Targeting FOSL1 suppresses miR-21-5p expression by interacting with the MIR21-SE. **(A)** ChIP-seq data revealed SE was formed around the MIR21 gene region and knockdown of FOSL1 suppressed the enrichment of MED1 and FOSL1 in the MIR21-SE. **(B, C)** Disruption of SE by JQ1 and iBET-151 inhibited the expression of miR-21-5p. **P< 0.01, ***P < 0.001 by one-way ANOVA. **(D–F)** The enrichments of MED1, BRD4 and FOSL1 in the MIR21-SE were eliminated in HNSCC cells upon JQ1 and iBET-151 treatment. **P < 0.01 by one-way ANOVA. **(G–I)** The enrichments of MED1 and FOSL1 in the MIR21-SE were eliminated in HNSCC cells treated with FOSL1 siRNA. **P < 0.01 by one-way ANOVA. **(J)** Knockdown of FOSL1 significantly suppressed the MIR21-SE luciferase activity. ***P < 0.001, **P<0.01 by one-way ANOVA. **(K)** MIR21-SE fragment was capable of elevating the luciferase reporter activity as compared to the negative control. **P < 0.01 by Student’s t-test **(L, M)** Knockdown of FOSL1 inhibited the expression of miR-21-5p. **P < 0.01, ***P < 0.001 by one-way ANOVA.

To dissect the upstream regulatory machinery of miR-21-5p, we tried to analyze its promoter and enhancer region of the *MIR21* gene based on our MED1 ChIP-seq results of human SCC cells from a different study (**Figure 2A**). Unexpectedly, we discovered that SEs were associated with *MIR21*. In agreement with ChIP-seq findings, RT-qPCR showed that the expression of miR-21-5p was decreased in SCC1 and HN6 cells upon JQ1 and iBET-151 treatment (**Figures 2B, C** **)**, two well-known BET inhibitors which can disrupt SE. Consistently, ChIP-PCR showed that the enrichments of MED1 and BRD4 on miR-21-SE region were also significantly suppressed in cells treated with JQ1 and iBET-151 (**Figures 2D, E** **)**. Interestingly, the enrichment of FOSL1 in promoter region of MIR21 was also decreased upon JQ1 and iBET-151 treatment (**Figure 2F**).

To further investigate the role of FOSL1 in establishment of the MIR21-SE, we analyzed the promoter and enhancer region of *MIR21* based on our FOSL1 ChIP-seq results of human SCC cells. Surprisingly, we found that FOSL1 was also significantly enriched in MIR21-SE region and MED1 occupancies on SEs in MIR21 were inhibited in cells treated with FOSL1 siRNAs (**Figure 2A**). ChIP-PCR results confirmed that the enrichments of MED1, BRD4 and FOSL1 on MIR21-SE region were significantly eliminated in cells treated with FOSL1 siRNA (**Figures 2G–I**). To further validate that FOSL1 can interact with the MIR21-SE, we cloned a fragment of MIR21-SE regions as well as a similar length negative control region into the pLG4.23 luciferase reporter. As shown in **Figure 2J**, knockdown of FOSL1 significantly suppressed the MIR21-SE luciferase activity. Consistently, the MIR21-SE fragment was also capable of elevating the luciferase reporter activity as compared to the negative control (**Figure 2K**). As expected, the expression of miR-21-5p was significantly inhibited in cells transfected with FOSL1 siRNA (**Figures 2L, M** **)**. These findings support a notion that FOSL1 promotes miR-21-5p expression by interacting with MIR21-SE.

### Restoration of miR-21 Attenuates FOSL1 Depletion-Mediated Inhibition of Cell Proliferation and Invasion in HNSCC Cells

**Figure 3:**
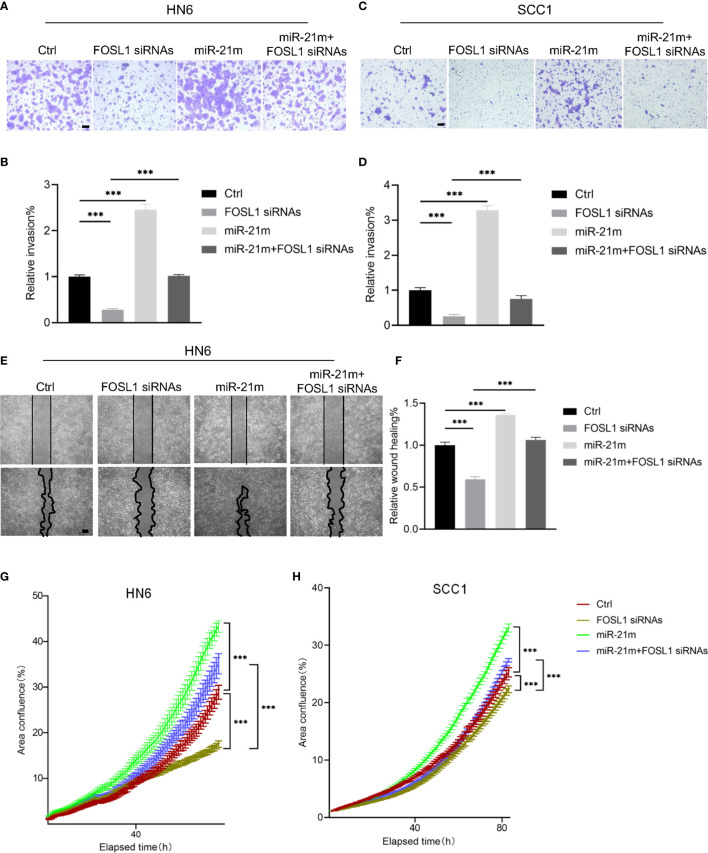
miR-21-5p was required for FOSL1-medaited malignant progression in HNSCC. **(A-D)** FOSL1 depletion-mediated inhibition of cell invasion was attenuated by overexpressing miR-21-5p in SCC1 and HN6 cells. ***P < 0.001 by one-way ANOVA. Scale bar, 200μm **(E, F)** FOSL1 depletion-mediated inhibition of cell migration was rescued by overexpressing miR-21-5p in HN6 cells. ***P < 0.001 by one-way ANOVA. Scale bar, 200μm **(G, H)** FOSL1 depletion-mediated inhibition of cell proliferation was impaired by overexpressing miR-21-5p in SCC1 and HN6 cells. ***P < 0.001 by two-way ANOVA.

To clarify the functional role of miR-21-5p in FOSL1-mediation cell proliferation and invasion, simultaneous knockdown of FOSL1 and restoration of miR-21-5p were performed in HNSCC cells. As shown in **Figures 3A–F**, FOSL1 depletion-mediated inhibition of cell invasion and migration were attenuated by overexpressing miR-21-5p. Similar results were also observed in cell proliferation assay (**Figures 3G, H** **)**. These findings indicate that miR-21-5p is involved in FOSL1-mediated invasion and proliferation in HNSCC *in vitro*.

### The Expression of miR-21-5p Is Positively Correlated With FOSL1 and Indicates Poor Prognosis in HNSCC

**Figure 4:**
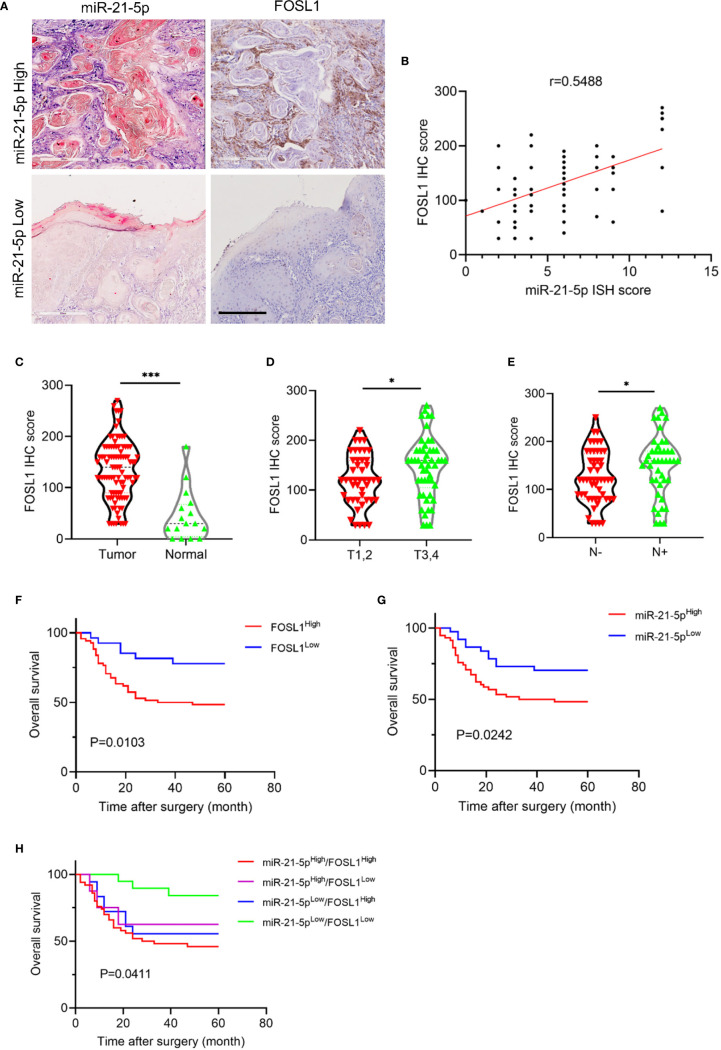
miR-21-5p expression was positively correlated with FOSL1 in HNSCC. **(A)** The representative images of miR-21-5p ISH staining and FOSL1 IHC staining. Scale bar, 300μm **(B)** FOSL1 expression was positively correlated with miR-21-5p expression. **(C)** The expression of FOSL1 was increased in HNSCC as comparted to the normal tissue. ***P < 0.001 by Student’s t-test **(D)** The expression of FOSL1 was increased in T3,4 stage HNSCC patient as comparted to those with T1,2 stage. *P < 0.05 by Student’s t-test **(E)** The expression of FOSL1 was increased in HNSCC patient with lymph node metastasis as comparted to those without lymph node metastasis. *P < 0.05 by Student’s t-test. **(F)** HNSCC patient with high FOSL1 expression levels indicated a poor overall survival. **(G)** HNSCC patient with high miR-21-5p expression levels indicated a poor overall survival. **(H)** HNSCC patient with high FOSL1 and miR-21-5p expression levels indicated a poorest overall survival.

Next, we analyzed the correlation of FOSL1 and miR-21-5p and then assessed their prognostic value in HNSCC. As shown in **Figures 4A, B**, FOSL1 expression was positively correlated with miR-21-5p expression in HNSCC, supporting the notion that FOSL1 promotes expression of miR-21-5p at the transcriptional level. As expected, the expression of FOSL1 was upregulated in HNSCC as compared to the normal tissue (**Figure 4C**). The increased expression of FOSL1 was also observed in T3,4 stage HNSCC when comparing to T1,2 stage HNSCC (**Figure 4D**). Similar results were also observed in HNSCC patients with lymph node metastasis as compared to those without lymph node metastasis (**Figure 4E**). The survival analysis revealed that high expression of miR-21-5p and FOSL1 indicated a poor prognosis in HNSCC (**Figures 4F, G** **)**. Moreover, HNSCC patients with high expression of miR-21-5p and FOSL1 showed the worst overall survival as compared to the other groups (**Figure 4H**).

## Discussion

MiR-21-5p has been extensively studied in variety of cancers and can function as an oncomiR to promote malignant progression of cancer, including HNSCC (25). As a result, miR-21-5p has been proposed as a promising diagnostic and prognostic biomarker, as well as an attractive therapeutic target for cancer treatment (25). However, the regulation of miR-21 is not well understood in HNSCC. Increasing evidences imply that miR-21 expression is maintained by transcriptional and post-transcriptional regulation (26, 27). Notably, it has been reported that AP-1, Ets/PU.1, C/EBPα, NFI, SRF, p53, STAT3 and AR binding sites were observed in the promoter region of the MIR21 gene, indicating that miR-21 expression is controlled by a transcriptional activator or suppressor (26, 28, 29). In agreement with these findings, our data shows that FOSL1, a member of the AP-1 family, occupied the promoter region of the MIR21 gene and regulated miR-21 expression. Moreover, FOSL1 is frequently dysregulated in HNSCC and has a critical role in the invasive growth, metastasis and stemness of HNSCC (30, 31). These results indicate that dysregulation of FOSL1 might exert its function by upregulation of miR-21-5p expression.

Recently, several studies implicating SEs have an important role in the regulation of ncRNAs, including miRNAs, circRNA and lincRNAs (32–34). As a new type of gene regulatory center, SEs are is often found to be positively correlated with oncogenes in cancer (35, 36). Strikingly, we found that a SE was formed around the MIR21 gene, which is enriched with FOSL1, indicating that miR-21 was controlled by FOSL1-associated SE in HNSCC. SEs are considered to be a large cluster of regulatory elements, which have a high binding capacity with transcriptional coactivators (such as BRD4, Mediator, CDK7 or EP300) as compared to typical enhancer binding, and SEs have high potential to activate their target gene transcription to control cell identity (37–40). To investigate the functional role of SEs in the regulation of miR-21-5p, JQ1 and iBET-151, two well-known BET inhibitors, were used to disrupt the SE (41). As expected, the expression of miR-21-5p was significantly suppressed in HNSCC cells upon treatment with JQ1 and iBET-151. Importantly, ChIP-qPCR data showed that the enrichments of MED1, BRD4 and FOSL1 were decreased in SE region of MIR21. To further demonstrate the role of FOSL1 in SE, we knocked-down the endogenous expression of FOSL1 in HNSCC cells, the ChIP-seq and ChIP-qPCR results revealed that MED1 and BRD4 enrichment on the MIR21-SE were also decreased. In agreement with these findings, the expression of miR-21-5p was significantly decreased in HNSCC cells treated with FOSL1 siRNA, supporting the notion that miR-21-5p was controlled by FOSL1 driven SE in HNSCC.

Taken together, we identified a SE associated with the MIR21 gene driven by FOSL1 in HNSCC, which uncovers a novel mechanism underlying miR-21-5p regulation in cancer.

## Data Availability Statement

The datasets presented in this study can be found in online repositories. The names of the repository/repositories and accession number(s) can be found below: https://www.ncbi.nlm.nih.gov/, GSM4567094 and GSM4567097.

## Ethics Statement

The studies involving human participants were reviewed and approved by Medical Ethics Committee of Hospital of Stomatology, Sun Yat-Sen University. The patients/participants provided their written informed consent to participate in this study.

## Author Contributions

CW and JL conceived the study and designed experiments. YW, RH, NX, WW, HC, MZ, GX, ZM, XX, XL, and ZH performed the *in vitro* experiment, immunohistochemical staining and clinical analysis. CW, JL, YW, and RH analyzed data, wrote, and edited the manuscript. All authors have discussed the results and provided comments on the manuscript for improving the manuscript. All authors contributed to the article and approved the submitted version.

## Funding

This work was supported by the National Natural Science Foundation of China (82073265, 81572661, 81772889), Guangdong Financial Fund for High-Caliber Hospital Construction (174-2018-XMZC-0001-03-0125/D-14), and Natural Science Foundation of Guangdong Province (2017A030313515, 2017A030313558).

## Conflict of Interest

The authors declare that the research was conducted in the absence of any commercial or financial relationships that could be construed as a potential conflict of interest.
